# Two-sided femoral *Campylobacter jejuni* osteomyelitis in a patient with acquired hypogammaglobulinemia: a case report

**DOI:** 10.1186/s12879-020-4929-8

**Published:** 2020-03-06

**Authors:** Joost Hartman, Matthijs Westerman, Jiri F. P. Wagenaar

**Affiliations:** 1 Internal Medicine, Northwest Clinics Alkmaar, Wilhelminalaan 12, Alkmaar, 1815 JD the Netherlands; 2 Internal Medicine, Hematology and Oncology, Northwest Clinics Alkmaar, Wilhelminalaan 12, Alkmaar, 1815 JD the Netherlands; 3 Internal Medicine and Infectious Diseases, Northwest Clinics Alkmaar, Wilhelminalaan 12, Alkmaar, 1815 JD the Netherlands

**Keywords:** Campylobacter, Osteomyelitis, Lymphoma, Hypogammaglobulinemia, Case report

## Abstract

**Background:**

*Campylobacter jejuni* is a motile, gram-negative rod known for causing self-limiting enterocolitis while rarely causing extraintestinal infections. We report the first case of a patient with *Campylobacter jejuni* osteomyelitis in both femora.

**Case presentation:**

A 54-year-old female presented with progressive pain in both upper extremities. Her past medical history mentioned a lymphoplasmacytic lymphoma (LPL) for which she had received dexamethasone, cyclophosphamide and fludarabine and was still receiving maintenance therapy with intravenous rituximab. Two months prior to presentation, she received oral fluoroquinolone for a recurrent enterocolitis with stool cultures positive for *Campylobacter jejuni*. Palpation of the left quadriceps femoris muscle was remarkably painful during physical examination. Laboratory testing showed elevated C-reactive protein and recent low gamma globulin levels. The presumptive diagnosis at this point was a transformation of LPL to a large B cell lymphoma. In order to determine the preferred site for biopsy, a fluorine-18 fluoro-2-deoxy-D-glucose positron emission tomography combined with computed tomography was done. However, blood cultures taken on admission showed growth of *Campylobacter jejuni* in both aerobic bottles, with a strain resistant to fluoroquinolones. Diagnosis of *Campylobacter jejuni* osteomyelitis was confirmed with 16S ribosomal RNA gene polymerase chain reaction performed on femoral bone obtained through biopsy. Treatment with intravenous imipenem/cilastatin followed by intravenous and oral doxycycline proved insufficient. Subsequently, the patient was treated successfully with intravenous meropenem for six weeks and concurrent intravenous immunoglobulin.

**Conclusion:**

We report the first case of *Campylobacter jejuni* osteomyelitis in both femora in a patient with acquired hypogammaglobulinemia. Diagnosis was confirmed by blood cultures and positive 16S ribosomal RNA gene polymerase chain reaction for *Campylobacter* spp. on bone biopsy. Treatment was successful with intravenous meropenem and immunoglobulin. Our report showcases an unusual manifestation in a patient with immunodeficiency and discusses failure of initial antibiotic therapy.

## Background

*C. jejuni* is a motile, gram-negative rod known for causing self-limiting enterocolitis while rarely causing extraintestinal infections. Bacteraemia is observed in less than 1% of affected patients with higher risk described in patients with immunodeficiency such as liver disease, hypogammaglobulinemia and human immunodeficiency virus infection [1, 2]. Osteomyelitis in an adult has only been described once [3]. We describe what we believe to be the first case of *C. jejuni* osteomyelitis occurring in both femora in a patient with an acquired hypogammaglobulinemia.

## Case presentation

A 54-year-old female presented with progressive pain in both upper extremities. Her past medical history mentioned a lymphoplasmacytic lymphoma (LPL), diagnosed three years prior to presentation, for which she had received intravenous rituximab, cyclophosphamide and oral dexamethasone. A relapse one year after diagnosis of LPL was treated with intravenous rituximab, fludarabine, and cyclophosphamide. Because of this relatively early relapse, rituximab (once every three months) was continued as maintenance therapy. Two months prior to presentation, she received oral antibiotic treatment for a recurrent enterocolitis with stool cultures positive for *C. jejuni*. The first culture showed no antibiotic resistance and the patient was treated with oral ciprofloxacin (500 mg twice daily for five days). Two subsequent cultures, taken because of remitting symptoms of diarrhoea, showed antibiotic resistance to fluoroquinolones and susceptibility to erythromycin and tetracycline. The patient was treated with oral azithromycin (500 mg once daily for three days) and diarrhoea subsided. Two weeks prior to presentation, polymyalgia rheumatica was suspected because of pain in upper extremities. In spite of empiric treatment with oral prednisolone (20 mg once daily for three days), pain persisted.

**Fig. 1:**
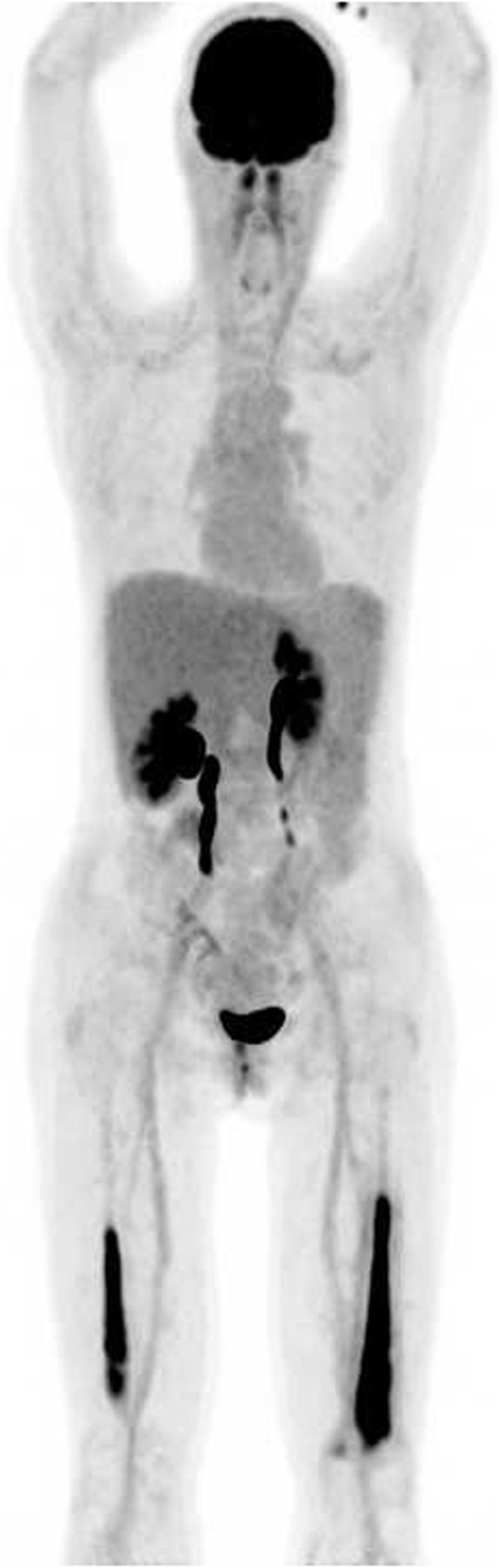
18 fluorodeoxyglucose-positron emission tomography/computed tomography showing intensified 18 FDG uptake in both femora

On presentation, the patient was afebrile and normotensive, with a heart rate of 80 beats per minute and an oxygen saturation of 99% on ambient air. Palpation of the left quadriceps femoris muscle was painful. Laboratory testing showed elevated C-reactive protein of 122 mg/L (normal value < 5.0 mg/L), erythrocyte sedimentation rate of 71 mm/hour (normal value < 20 mm/hour), and normal red cell count and white blood cell differential count. Levels of calcium, alkaline phosphatase, creatine kinase, and lactate dehydrogenase were within normal range. Recent gamma globulin levels were low: IgM < 0.05 g/L (normal range 0.40–2.30 g/L), IgA 0.12 g/L (normal range 0.70–4.00 g/L) and IgG 4.2 g/L (normal range 7.0–16.0 g/L) without presence of a monoclonal gammopathy. Differential diagnosis consisted of transformation of LPL to a large B cell lymphoma, or osteomyelitis. A fluorine-18 fluoro-2-deoxy-D-glucose (F-18 FDG) positron emission tomography combined with computed tomography (PET/CT) was done to determine the preferred site for biopsy. Images showed increased FDG uptake in both distal femora (Fig. 1).

A biopsy of the left femur was performed and histology was negative for B lymphocytes, but showed necrotic and fibrous tissue with T lymphocytes and macrophages in clusters. CD20 immunostaining was negative. Meanwhile, blood cultures taken on admission showed growth of *C. jejuni* in both aerobic bottles (BacT/ALERT), with a strain resistant to fluoroquinolones and susceptible to tetracyclines and carbapenems. The diagnosis *C. jejuni* osteomyelitis was confirmed with positive 16S ribosomal RNA gene polymerase chain reaction of *Campylobacter* spp. on prior femoral bone biopsy. Treatment was started with intravenous imipenem combined with cilastatin (500 mg / 500 mg four times daily) and concurrent intravenous immunoglobulin (IVIG) Privigen® (20,000 mg once monthly). Rituximab was discontinued. After two days, intravenous imipenem/cilistatin was substituted for intravenous doxycycline (100 mg twice daily). Because of a suspected sufficient oral bioavailability and bone penetration, oral doxycycline (100 mg twice daily) was started after four days of intravenous antibiotic treatment. However, blood cultures taken after eight days of oral doxycycline were again positive for *C. jejuni* with a cultured strain susceptible to imipenem, meropenem, erythromycin and tetracycline. Femoral pain recurred. A transoesophageal echocardiography showed no vegetation, making an endocarditis less likely. Intravenous doxycycline was started and after three days, blood cultures were positive again with unchanged susceptibility. When therapy with intravenous meropenem (1000 mg three times daily) was started, pain in upper extremities subsided and blood cultures on follow-up were negative. Antibiotic treatment was continued intravenously for a total duration of six weeks. The patient was doing well on follow-up one year later.

## Discussion and conclusion

An enterocolitis caused by *C. jejuni* is a common occurrence, but osteomyelitis is a rare manifestation and has not been previously described in both femora. Immunocompetent individuals usually resolve an enterocolitis without antibiotic treatment, for which both innate and humoral immune response seem to be important in clearance of bacteria. Infected patients with *Campylobacter* spp*.* develop specific immunoglobulin IgG and IgM in serum, and IgA antibodies in both serum and intestinal secretions. IgA is considered the first line of humoral defence in the intestinal tract against a bacteraemia with *C. jejuni*, after which IgM and IgG can provide second line and more specific humoral defence in an immunocompetent patient [1]. Patients with an immunoglobulin deficiency often develop prolonged, severe, and recurrent *C. jejuni* infections, and have an increased risk of bacteraemia [2]. Treatment with IVIG is an option in case of hypogammaglobulinemia, but the added effect of IVIG has not been studied specifically in *Campylobacter* spp*.* infections and its use is therefore debatable [4, 5]. There have been reports of patients treated with intravenous IgM who had better bacterial clearance than with predominantly IgG containing IVIG [6], but no randomized controlled studies have been performed. Although hypogammaglobulinemia in our patient persisted after cessation of rituximab, we decided to stop treatment with IVIG eight months after successfully treating the osteomyelitis. Gamma globulin levels during follow-up remained low.

Due to its rarity, no specific guidelines for the treatment of *C. jejuni* osteomyelitis exist. The pathophysiological mechanism of bacterial osteomyelitis can be divided into three categories: by hematogenous spread, by a contiguous focus, and by vascular insufficiency. Hematogenous osteomyelitis usually affects the metaphysis of long bones because of slowing of blood in the vascular loops which can cause deposition of microbes. Inflammation can lead to increased pressure in the medullary bone, which can cause the infection to break through the cortex and periosteum. This can ultimately cause bone necrosis [7]. Current expert opinion on treatment for bacterial osteomyelitis consists of parenteral or highly available oral antimicrobial therapy for a duration of four to six weeks, and sometimes surgical management is necessary [3, 7]. In case of a bacteraemia with *Campylobacter* spp*.*, resistance to fluoroquinolones is increasingly reported with percentages as high as 50% of isolates in blood cultures and a patient mortality of 16.4% [2].

In our patient, it was hypothesized that oral ingestion of *C. jejuni* led to intestinal colonization. Unclear is when and how this occurred. Serum IgA, IgG, and IgM were deficient because of an acquired deficiency of antibody-mediated immunity due to rituximab induced cell death of B cells, and because of a medical history of LPL with corresponding chemotherapy. Because of a recurrent enterocolitis and IgA deficiency, it is likely that bacteraemia occurred. It is suspected that the deficiency in serum IgM and IgG increased her risk of bacterial seeding and led to an osteomyelitis. Unclear is why hematogenous bacterial seeding to both femora occurred. F-18 FDG PET/CT showed no aortitis or mycotic aneurysm. Although the patient reported no prior pain in lower extremities, our theory is that: 1) there might have been avascular necrosis after systemic corticosteroid therapy. This theory is supported by histological findings, although the femoral head was not involved in our case [8]; 2) past bone marrow involvement of LPL in both femora caused a susceptibility for bacterial seeding in bone. Failing of oral therapy with doxycycline could possibly be explained by its bacteriostatic properties and by poor intestinal absorption during an enterocolitis. We also suspect inadequate bone penetration of doxycycline in our patient because three days of intravenous therapy did not result in reduction of pain and blood cultures remained positive. Reports about bone penetration of doxycycline show contrasting results, whereas bone penetration of carbapenems is uniformly reported as adequate [9, 10].

In conclusion, we describe the first case of a patient with *C. jejuni* osteomyelitis occurring in both femora. An acquired hypogammaglobulinemia increased her risk of sustained bacteraemia and hematogenous bacterial seeding to both femora. Diagnosis was made by positive blood cultures and positive 16S ribosomal RNA gene polymerase chain reaction on bone biopsy. Treatment with intravenous imipenem/cilastatin followed by intravenous and oral doxycycline proved insufficient. The patient was successfully treated with intravenous meropenem for six weeks and concurrent IVIG. Carbapenems may offer an effective treatment option for osteomyelitis with *C. jejuni* due to reported bone penetration, its bactericidal properties, and current susceptibility patterns. Concurrent treatment with IVIG can be considered.

## Data Availability

Not applicable.

## Abbreviations

F-18 FDG PET/CT: Fluorine-18 fluoro-2-deoxy-D-glucose positron emission tomography combined with computed tomography
IVIG: Intravenous immunoglobulin
LPL: Lymphoplasmacytic lymphoma
